# Evaluating efficacy and safety of sub-anesthetic dose esketamine as an adjuvant to propofol/remifentanil analgosedation and spontaneous respiration for children flexible fibreoptic bronchoscopy: a prospective, double-blinded, randomized, and placebo-controlled clinical trial

**DOI:** 10.3389/fphar.2023.1184663

**Published:** 2023-05-09

**Authors:** Yu Zhong, Min Jiang, Yunshi Wang, Tingting Su, Yuanzhi Lv, Zhiqing Fan, Hengyi Ning, Yilan Yang, Yanhua Chen, Yubo Xie

**Affiliations:** ^1^ Department of Anesthesiology, The First Affiliated Hospital of Guangxi Medical University, Nanning, Guangxi, China; ^2^ Department of Paediatrics, The First Affiliated Hospital of Guangxi Medical University, Nanning, Guangxi, China; ^3^ Department of Anesthesiology, Cardiovascular Institute, Nanning, Guangxi, China

**Keywords:** esketamine, sub-anesthetic dose, spontaneous respiration, flexible fiberoptic bronchoscopy in children, propofol, remifentanil

## Abstract

**Background:** Flexible fiberoptic bronchoscopy (FFB) for children is widely performed under sedation. Currently, the optimal sedation regimen remains unclear. Esketamine is an N-methyl-D-aspartic acid (NMDA) receptor antagonist, which has stronger sedative and analgesic effects and exerts less cardiorespiratory depression than other sedatives. The purpose of this study was to evaluate whether a subanesthetic dose of esketamine as an adjuvant to propofol/remifentanil and spontaneous ventilation compared with control reduces the procedural and anesthesia-related complications of FFB in children.

**Materials and methods:** Seventy-two children ≤ 12 years of age who were scheduled for FFB were randomly assigned, in a 1:1 ratio, to the esketamine-propofol/remifentanil (Group S, *n* = 36) or to the propofol/remifentanil group (Group C, *n* = 36). All children were retained spontaneous ventilation. The primary outcome was the incidence of oxygen desaturation (respiratory depression). Perioperative hemodynamic variables, blood oxygen saturation (SPO_2_), end-tidal partial pressure of carbon dioxide (PetCO_2_), respiratory rate (R), and the Bispectral index (BIS), induction time, procedural time, recovery time, the time to the ward from the recovery room, consumption of propofol and remifentanil during the procedure and the appearance of adverse events, including paradoxical agitation following midazolam administration, injection pain, laryngospasm, bronchospasm, PONV, vertigo, and hallucination were also compared.

**Results:** The incidence of oxygen desaturation was significantly lower in Group S (8.3%) compared to Group C (36.1%, *p* = 0.005). The perioperative hemodynamic profile including SBP, DBP, and HR were more stable in Group S than that in Group C (*p* < 0.05). Consumption of propofol and remifentanil was lower in Group S than in Group C (*p* < 0.05). Furthermore, PAED scores, cough scores and injection pain were lower in the Group S than in Group C (*p* < 0.05). The recovery time of Group S was slightly longer than that of Group C (*p* < 0.05). Nobody happened paradoxical agitation following midazolam administration, PONV, vertigo, and hallucinations in both groups (*p* > 0.05).

**Conclusion:** Our findings indicate that a subanesthetic dose of esketamine as an adjuvant to propofol/remifentanil and spontaneous respiration is an effective regimen for children undergoing FFB. Our findings will provide a reference for clinical sedation practice during these procedures in children.

**Clinical Trail Registration:** Chinese clinicaltrials.gov registry (identifier: ChiCTR2100053302).

## Introduction

Flexible fiberoptic bronchoscopy (FFB) is an important endoscopic procedure that has been performed primarily for the diagnosis and management of a variety of lung conditions and respiratory disorders (Strohleit et al., 2021). This invasive operation can cause severe cough, airway spasm, and hemodynamic fluctuations, which may seriously threaten the safety of the lives of children (Mondal et al., 2018). Ideally, it is performed under deep sedation or general anesthesia, with the former being used more often for this procedure in children (Huang et al., 2022). Compared to general anesthesia, deep sedation can maintain spontaneous breathing, provides a better ventilation/blood flow ratio, effective alveolar ventilation, and no barotrauma due to significant mechanical ventilation, reduced side effects, and ultimately shortens the duration of hospitalization (Bhat et al., 2019; Murgu et al., 2020). Nonetheless, the best sedation regimen to facilitate children undergoing FFB has not been clearly established.

Procedural sedation has been achieved with propofol alone or propofol combined with different adjuncts in pediatric patients (Fuentes et al., 2018). One of the main anesthesia schemes to achieve painless FFB is that of propofol anesthesia supplemented with ultra-short-acting opioid drugs, remifentanil, for children (Tschiedel et al., 2021; Zha et al., 2021). The clinical characteristics of propofol and remifentanilis are rapid effect, short duration, and rapid recovery. However, propofol has a narrow therapeutic window for children, and the administration of propofol can cause dose-dependent adverse events, including oxygen desaturation, loss of protective reflexes of the airways, apnea, obstruction of the upper airways, painful injection, severe hypotension and bradycardia (Watt et al., 2016). Infusion of remifentanil can significantly slow the respiratory rate, cause respiratory depression (Lee et al., 2019). Consequently, a new drug is needed to decrease the incidence of complications.

Esketamine, the S-enantiomer of ketamine racemate, acts as a new N-methyl-D-aspartic acid (NMDA) receptor antagonist, it has approximately two times higher sedative potency compared to racemic ketamine (Huang et al., 2022). Esketamine offers many advantages as an adjuvant, including maintaining airway tone and hemodynamic stability, and serves as an ideal choice for anesthesia and sedation (Trimmel et al., 2018). However, Brinck et al. revealed that increased or repeated doses of esketamine can lead to psychotomimetic effects, increased respiratory secretion, although esketamine secretion is lower than that of ketamine, which prolongs recovery time, and induction of vomiting, tachycardia, high blood pressure, and other adverse reactions (Brinck et al., 2018). The subanesthetic dose refers to a dose lower than that of the anesthetic drug, and can play a role in enhancing anesthesia. In recent years, subanesthetic doses of esketamine as adjuvant has been shown to be feasible and can effectively lower the intraoperative dose of propofol and opioids, and beneficial for rapid recovery of patients (Chen et al., 2022).

Currently, there are limited data on the efficacy and safety of esketamine at subanesthetic dose as an adjuvant to propofol/remifentanil analgosedation and spontaneous respiration for children undergoing FFB. Therefore, our objective in this study was to evaluate the efficacy and safety of as a subanesthetic dose esketamine combined with propofol/remifentanil compared to the propofol/remifentanil regimen alone during FFB.

## Materials and methods

### Ethics approval and consent to participate in the study

This study was approved by the Ethics Committee of the First Affiliated Hospital of Guangxi Medical University (Ethics approval number: No. 2021 (KY-E-261), Head: Prof. Dr. Zhong), registered in the Chinese Clinical Trials Registry (Chinese Clinical Trial Registry: https://www.chictr.org.cn/showproj.aspx?proj=139609. Registration number: ChiCTR2100053302. Principal Investigator: Zhong Yu) and was conducted in accordance with the International Conference on Harmonization Guidelines for Good Clinical Practice and the Declaration of Helsinki. We obtained written informed consent of the parents or guardians of all participants in this study.

### Study patients

Pediatric patients aged ≤ 12 years scheduled to undergo elective bronchoscopy were selected as study objects, with the American Society of Anesthesiologists (ASA) status classification of I–III. All families of children agreed to participate in the study and provided signed informed consent. Exclusion criteria included parents who refused to participate in the study, previous history of heart disease, cognitive or central nervous system disease, severe liver function, renal insufficiency (prothrombin ratio less than 15%), history of severe hypertension, allergy to anesthesia drugs and abuse of sedative drugs, a high risk of increased intracranial pressure, glaucoma, other complications associated with increased intraocular pressure, and poor treatment of hyperthyroidism.

### Randomization and grouping

In this study, all children were assigned sequential inclusion numbers. The children were randomly assigned in a 1:1 ratio to two groups. Group S received intravenous anesthesia with esketamine combined with propofol/remifentanil and group C received intravenous anesthesia with propofol/remifentanil. The sealed envelopes were numbered, and each envelope contained the corresponding random number and the anesthesia protocol to be applied. Allocation was kept strictly confidential. The children were enrolled according to inclusion and exclusion criteria before the procedure. Serial numbers were obtained using a computerized random number generator with a permuted block randomization scheme. The corresponding serial number envelope was removed to provide the anesthesia protocol on the day of the procedure. An assistant not involved in the FFB procedure prepared the drugs. The anesthesiologist performed the anesthesia according to the instructions in the envelope. Children, bronchoscopists, and anesthesiologists, nurses, data collectors, and statistical analysts were blinded to the group allocation.

### Study protocol

This was a prospective, double-blind, randomized, and placebo-controlled clinical trial conducted at the Department of Anesthesiology, the First Affiliated Hospital of Guangxi Medical University. The time of the first enrolled patient from 18 March 2022 to the time of the last enrolled patient to 19 August2022.

Two professional bronchoscopists performed FFB for children. One is the chief physician and the other is the associate chief physician in the Department of Pediatrics at our hospital. They used a flexible bronchoscope (BF-XP290, BF-P290, Olympus, Tokyo, Japan) introduced using the nasal approach with subjects in the supine position. The indications for bronchoscopy included pneumonia, interstitial lung disease, bronchial asthma and bronchiectasis. Pediatricians were allowed to decide which specific type of bronchoscopy procedures to use according to the child’s pulmonary disease, medical imaging, and other auxiliary examination analysis. Types of procedures included bronchoalveolar lavage (BAL), bronchial brushing (BB), transbronchial lung biopsy (TBLB), and endobronchial biopsy (EBB). Bronchoalveolar lavage (BAL) was performed using 0.5–1 mL/kg (maximum 5 mL/kg total) aliquots of nonbacteriostatic 0.9% saline.

Bronchoscopy was performed in the outpatient bronchoscopy room. The anesthetic technique was standardized as follows: all children were fasted for 2 h for clear liquids, 4 h for breast milk, and 6 h for infant formula, non-human milk, and light meal before the procedure, and venous access was performed and intravenous atropine 10 μg/kg was administered prior to the operation. Midazolam was administered intravenously (0.05 mg/kg) as premedication to relieve significant preprocedural anxiety. During sedation, all children received oxygen via a face mask at 6 L/min. Heart rate (HR), systolic blood pressure (SBP), diastolic blood pressure (DBP), blood oxygen saturation (SPO_2_), respiratory rate (R), end-tidal partial pressure of carbon dioxide (PetCO_2_) and Bispectral index (BIS) values were monitored. Group S received the loading dose of esketamine 0.3 mg/kg and propofol 2–2.5 mg/kg by intravenous injection, a loading dose of propofol and esketamine was administered for 3 min to avoid apnea. Next, esketamine 0.3 mg/kg/h, propofol 4–10 mg/kg/h and remifentanil 0.05–0.3 μg/kg/min were administered by continuous infusion. Referring to the study by Xia et al. (Shen et al., 2012), the remifentanil infusion was titrated at an initial rate of 0.05 μg/kg/min to decrease the respiratory rate to 50% of the baseline value in increments of 0.05 μg/kg/min with reference to the respiratory rate before the procedure as the baseline value, anesthesiologists were instructed to increase or decrease the propofol infusions, in steps of ± (1–2) mg/kg/h. Group C received the same regimen as Group S, but esketamine was replaced by normal saline with the same volume. Children in both groups retained spontaneous breathing during the procedure. The BIS monitor (Covidien, United States), consisting of four electrodes placed on the forehead of the children, was used to monitor the continuous electroencephalogram (EEG) recording of the children. The BIS was processed as a dimensionless number between 0 and 100 from a frontal electroencephalographic signal. The fiberoptic bronchoscope was introduced through the mask of the soft adhesive cap of the T-type connector tube, through the glottis, carina, and left and right main bronchus. Surface anesthesia was achieved by locally spraying with 1% lidocaine hydrochloride (maximum 7 mg/kg) through the mouth spray unit of the bronchoscope biopsy device during the procedure. When the BIS reached 55–65, the children no longer presented any obvious physical activity and the eyelash reflex disappeared, and an endoscopic examination was started. The rate of administration of remifentanil was adjusted throughout the procedure to maintain the target respiratory rate mentioned above. Propofol infusion was titrated to a clinically adequate depth of anesthesia according to the BIS value. Propofol (0.5–1 mg/kg) was injected intravenously to increase the depth of anesthesia when coughing and body movement occurred during the procedure. For children presenting transient episodes of SpO_2_ below 92%, the treatment measures were to stop drug administration. High fresh oxygen was supplied first to obtain sufficient ventilation and compensate for airway leaks and oxygen delivery was then increased from 6 to 10 L/min. If this did not work, we provided mandibular support to the children. If none of these approaches were successful, the bronchoscopists removed the bronchoscope and ventilated the face mask over the patient for a few minutes. If obstruction or desaturation was not resolved, children received the appropriate airway intervention (oropharyngeal airway, nasopharyngeal ventilation, laryngeal mask airway, tracheal intubation, or positive pressure ventilation assistance) until the SpO_2_ increased above 95%, and then the procedure was started again stepwise as needed. Perioperative PetCO_2_ was used as a monitor via a nasal catheter in both groups. As it was difficult to collect PetCO_2_ from the children before sedation, 40 mmHg was used as the baseline value of PetCO_2_. All medications were stopped immediately at the end of the bronchoscopy. Children were transferred to the recovery room with oxygen support to maintain oxygen saturation at least above 92%. Recovery times, the time to the ward from the recovery room, cough scores, PAED scores, and adverse events were also recorded. Children were transferred to the ward from the recovery room once they were awake and had regained their orientation, and the Aldrete wake-up score was 9 points.

### Primary outcome

The primary endpoint of the study, the efficacy of the anesthetic regimen of Group S *versus* Group C on the incidence of intraoperative hypoxemia is the rate of SpO_2_ <90% lasting 10 s during bronchoscopy. The severity of hypoxemia was divided into three levels: mild (SpO_2_: 80%–89%), moderate (SpO_2_: 70%–79%), or severe (SpO_2_: < 70%) (Bakan et al., 2014). All episodes of apnea and the required interventions were documented.

### Secondary outcomes

Secondary outcomes included perioperative hemodynamic variables (BP, HR), SPO_2_, R, BIS of the two groups, which were compared before anesthesia induction (T_0_), after anesthesia induction (T_1_), when the fiberoptic bronchoscopy reached the glottis (T_2_), when alveolar lavage was performed (T_3_), immediately after the end of bronchoscopy (T_4_) and 10 min after the end of bronchoscopy (T_5_). The PetCO_2_ of the two groups was analyzed before anesthesia induction (T_0_), after anesthesia induction (T_1_), immediately after the end of bronchoscopy (T_0min_), 5 min after the end of bronchoscopy (T_5min_), and 10 min after the end of bronchoscopy (T_10min_). The induction time (from the injection of the medication to the disappearance of the eyelash reflex and no obvious physical activity after laryngoscopy served as an indicator), procedure time (the time from introduction of the bronchoscope until its removal), consumption of propofol and remifentanil during the operation were recorded. After the bronchoscopy, the children were moved to the recovery room. In the recovery room, the Pediatric Anesthesia Emergence Delirium (PAED) scores, cough scores, the recovery time (the time after the bronchoscopy procedure ended until full consciousness) and the time to the ward from the recovery room were also noted. The PAED of two groups were recorded when the children had fully emerged from anesthesia (time 0) and every 5 min thereafter, up to 30 min thereafter, and the highest score was the effective value. The PAED scale included five items: eye contact with the nurse, action according to instructions, attention to the surrounding environment, anxiety, agitation, crying, and difficulty appeasing. The score for each item was 0–4 points and the total score was 0–20 points. The higher the total score, the more agitated the child was. PAED scores ≥ 10 were considered to define agitation (Simonsen et al., 2020). When the child completed the examination and was fully recovered from anesthesia, the cough score was recorded, and was recorded every 5 min until 30 min had elapsed. The severity of the cough was classified according to the number of episodes of cough. No coughing was defined as grade 0, once or twice coughing was defined as grade 1, 3–4 times coughing was defined as grade 2, 5 or more times coughing was defined as grade 3. A cough score ≥ 2 was classified as having a severe cough (Ozturk et al., 2016). Adverse events such as paradoxical agitation following midazolam administration, propofol injection pain, incidence of PONV, airway spasm, vertigo, and hallucinations were also recorded. A four-point pain scale was used to continually evaluate the severity of pain from the propofol injection during anesthesia induction for all children (Guan et al., 2021). Briefly, grade 0 indicated that there is no pain (negative response to questioning), mild pain (pain reported in response to questioning, without behavioral signs) was defined as grade 1, moderate pain (pain reported in response to questioning and accompanied by a behavioral sign or pain reported spontaneously) was defined as grade 2, and severe pain (strong vocal response or response accompanied by facial grimacing, arm retraction, or tears) was defined as grade 3.

### Sample size

The sample size estimated for this trial was based on the results of the preliminary experimental results for this study and on clinical judgment. The required sample size was calculated for the primary outcome and the incidence of perioperative hypoxemia. In a preliminary study, the incidence of oxygen desaturation in Group C was 40%, with a reduction from 40% to 10% considered to be of clinical importance (*α* = 0.05, power = 0.8). The analysis showed that 32 subjects per group would be sufficient to detect a statistical difference between the two groups. Given the 10% dropout rate, a total of 72 pediatric patients (*n* = 36 per group) were required to achieve a level of significance, respectively, in this study. The G-power software was used to calculate the sample size (version V3.1.9.3).

### Statistical analysis

All data are expressed as mean ± standard deviation, interquartile range, or numbers (%). Continuous variables with normal distribution (weight, HR, SBP, DBP, BIS, duration of induction, duration of procedure, duration of anesthesia, recovery time, and the time to the ward from the recovery room) were presented as mean ± SD, and continuous variables with non-normal distribution (age, height, RR, SpO_2_, PetCO_2_, dose of propofol and remifentanil, number of times propofol was added, PAED score, and cough score) were represented by median (interquartile range) and the 25th and 75th percentile (p25, p75). Frequency (%) was used for categorical variables (sex, ASA score, FB indication, FB procedures, desaturation, airway assistance steps, paradoxical agitation following midazolam administration, injection pain, laryngospasm, bronchospasm, PONV, vertigo, and hallucination). Normally distributed data between the two groups were assessed using the Student’s t-test, and variables at different time points within each group were compared by repeated measure ANOVA. Continuous variables with non-normal distributions were compared between the two groups using the Wilcoxon rank sum test. Categorical data were compared using the *x* ^2^ test or Fisher’s test, as appropriate. All statistical analyses were performed with IBM SPSS v25.0 statistical software and GraphPad Prism (version 9.5.0). A *p*-value or corrected *p*-value of < 0.05 were defined as statistically significant.

## Results

### Patient inclusion and demographic characteristics

**FIGURE 1:**
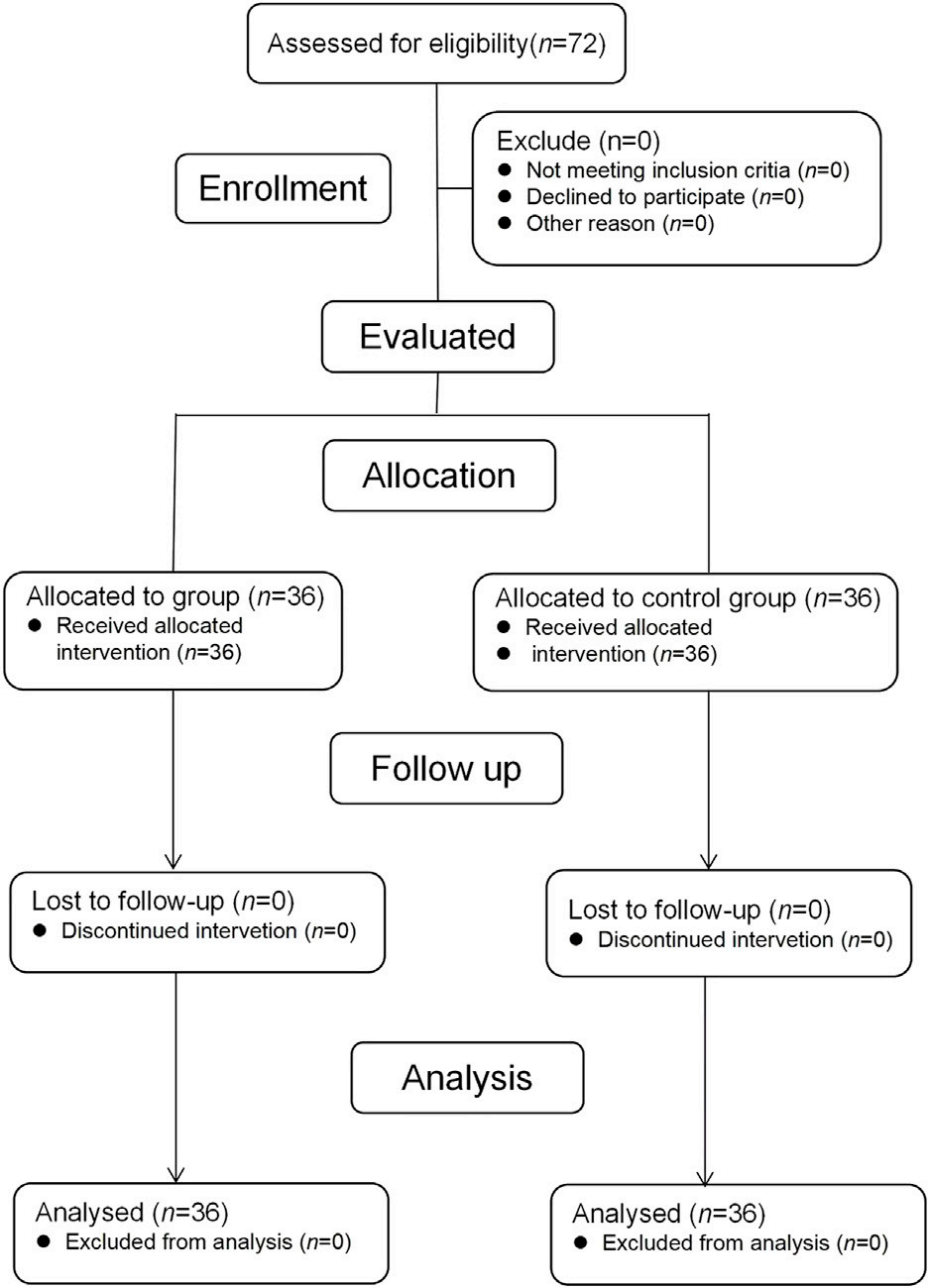
Flow diagram of participant enrollment. Abbreviations: Group S, esketamine-propofol/remifentanil group; Group C, propofol/remifentanil group.

A total of 72 pediatric patients were enrolled and completed the study (Figure 1). There were no significant differences between groups in terms of age, sex, weight, height, ASA score, indications for FB and the specific type of bronchoscopy procedures (Table 1).

**TABLE 1:** Characteristics of two patient groups.

Parameter	Group C	Group S	*p* value
Age	8 (4.25, 10)	6.5 (4, 9)	0.194
Gender (F)			
Female (*n%*)	16 (44%)	16 (44%)	1.0
Male (*n%*)	20 (55.6%)	20 (55.6%)	1.0
Weight (kg)	22.86 ± 7.12	20.41 ± 9.00	0.204
Hight (cm)	123 (112,135)	122 (108,139)	0.860
ASA score (*n%*)			
Ⅰ	12 (33.3%)	10 (27.8%)	0.609
Ⅱ	23 (63.9%)	25 (69.4%)	0.617
Ⅲ	1 (2.8%)	1 (2.8%)	1.0
Indications for FB (*n%*)			
pneumonia	15 (41.7%)	13 (36.1%)	0.629
Interstitial lung disease	5 (13.9%)	8 (22.2%)	0.358
Bronchial asthma	4 (11%)	4 (11%)	1.0
Bronchiectasis	1 (2.8%)	0 (0)	1.0
Others	11 (31%)	11 (31%)	1.0
Procedures for FB (*n%*)			
BAL	11 (30.6%)	13 (36.1%)	0.617
BAL + TBLB	5 (13.9%)	8 (22.2%)	0.358
BAL + BB	9 (25%)	7(19.4%)	0.571
BAL + EBB	11 (30.6%)	8 (22.2%)	0.422

Data are displayed as the mean ± SD or number of cases. One-way analysis of variance was used to analyze the body weight in two groups. Age, height was continuous variables with non-normal distribution and represented by median (interquartile range) and 25th and 75th percentile (p25, p75). Frequency (%) was used for categorical variables (gender, ASA score, indication for FB, procedures for FB). No statistically significant differences were observed between the groups.

Abbreviations: Group S, esketamine-propofol /remifentanil group; Group C, propofol /remifentanil group; ASA score, American Society of Anesthesiologists score; BAL, bronchoalveolar lavage; BAL + TBLB, bronchoalveolar lavage and transbronchial lung biopsy; BAL + BB, bronchoalveolar lavage and bronchial brushing; BAL + EBB, bronchoalveolar lavage and endobronchial biopsy.

### Perioperative hypoxemia

Table 2 shows the incidence of oxygen desaturation and the steps of airway assistance. All children successfully completed the FFB procedure. The incidence of oxygen desaturation was lower in Group S compared to Group C (*p* = 0.005). Seven children in Group C and three children in Group S experienced mild desaturation. While six children in Group C experienced moderate desaturation, respectively, during the procedure (*p* = 0.025). Ten children in Group C and three children in Group S recovered from oxygen desaturation after mandibular support (*p* = 0.032), and three children in Group C recovered with a face mask and manual ventilation. The episodes of desaturation were short in duration. Neither group required tracheal intubation and mechanical positive pressure ventilation.

**TABLE 2:** Incidence of desaturation and need for airway assistance.

	Group C	Group S	*p*-value
Desaturation (*n%*)	13(36.1%)	3(8.3%)	0.005
mild (SpO_2_: 80–89%)	7(19.4%)	3(8.3%)	0.173
moderate(SpO_2_:70–79%)	6(16.7%)	0	0.025
severe (SpO_2_: 70%)	0	0	-
Steps of airway assistance			
Increase in oxygen flow	0	0	-
mandibular support	10(27.8%)	3(8.3%)	0.032
Face mask and manual ventilation	3(8.3%)	0	0.239
Tracheal intubation and positive-pressure ventilation	0	0	-

Values are given as number of subjects (%). Chi-square tests or Fisher's exact test was used to analyze the incidence of desaturation.

Abbreviations: Group S, esketamine-propofol/remifentanil group; Group C, propofol/remifentanil group.

### Hemodynamic variables, R, PetCO_2_, and BIS value

**FIGURE 2:**
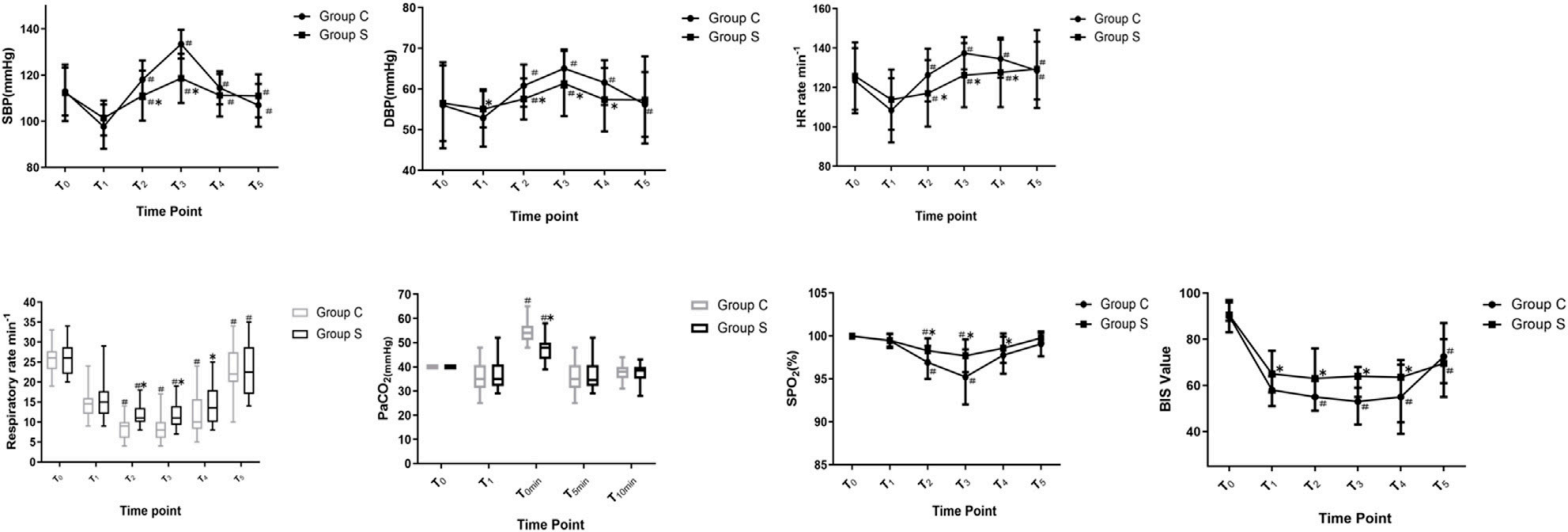
Changes in Hemodynamic variables, R, PetCO_2_ and BIS value of patients. Compare with T_1_, ^#^ *p* < 0.05; compare with Group C, *p < 0.05. Abbreviations: SBP, systolic blood pressure; DBP, diastolic blood pressure; HR, heart rate; R, respiratory rate; SPO_2_, pulse oximetry; PetCO_2_, end-tidal carbon dioxide of pressure; BIS, bispectral index. Group S, esketamine-propofol/remifentanil group; Group C, propofol/remifentanil group.

There were no significant differences in SBP, DBP, HR, SpO_2_, R, PetCO_2_, or BIS for the two groups at T_0_ (Figure 2, *p* > 0.05). Compared to T_1_, SBP increased in T_2_–T_5_ in two groups (*p* < 0.05). DBP increased at T_2_–T_3_ in Group S, and DBP increased at T_2_–T_5_ in Group C (*p* < 0.05). HR increased at T_2_–T_5_ in two groups (*p* < 0.05). R was slowed at T_2_ and T_3_ and increased at T_5_ in Group S (*p* < 0.05), while R was slowed at T_2_–T_4_ and increased at T_5_ in Group C (*p* < 0.05). SPO_2_ decreased at T_2_–T_3_ in two groups (*p* < 0.05), and PetCO_2_ in Group S and Group C increased at T_0min_ (*p* < 0.05). In Group C, the BIS value decreased at T_2_–T_4_ (*p* < 0.05) and increased at T_5_ (*p* < 0.05). In Group S, the BIS value increased at T_5_ (*p* < 0.05). Compared with Group C, the SBP in Group S decreased at T_2_–T_3_ (*p* < 0.05), DBP and HR decreased at T_2_–T_4_ (*p* < 0.05), R and SPO_2_ increased at T_2_–T_4_ (*p* < 0.05), PetCO_2_ decreased at T_0min_ (*p* < 0.05), and the BIS value increased at T_1_–T_4_ (*p* < 0.05).

### Evaluation of anesthesia-related indices and drug consumption

Comparison of induction time duration, duration of the procedure, duration of anesthesia, recovery time, the time to the ward from the recovery room, consumption of propofol and remifentanil drugs, number of times propofol was added between the two groups is summarized in Table 3. There were no significant differences in terms of duration of procedure, anesthesia, and the time to the ward from the recovery room between the two groups. Compare with Group C, the duration of induction was shorter in Group S (*p* = 0.02). The recovery time of Group S was longer than that of Group C (*p* = 0.001). Drug consumption of propofol (*p* = 0.013) and remifentanil (*p* = 0.001) in Group S was less than that in Group C, and the number of propofol additions in Group S was less than that in Group C (*p* = 0.001).

**TABLE 3:** | Duration of induction time, duration of procedure, duration of anesthesia, recovery time, The time to the ward from the recovery room, dose of propofol and remifentanil, number of times propofol was added between the two groups.

Parameter	Group C	Group S	*p*-value
Duration of induction (min)	10.10 ± 1.03	9.56 ± 0.89	0.02
Duration of procedure (min)	14.85 ± 6.65	15.39 ± 5.82	0.192
Duration of anesthesia (min)	24.06± 6.17	24.36 ± 6.07	0.328
Recovery time (min)	13.01 ± 2.05	15.06 ± 1.65	0.001
The time to the ward from the recovery room (min)	49.13 ± 4.67	49.78 ± 4.35	0.590
Dose of propofol (mg)	131 (96,176)	119 (82,170)	0.013
Dose of remifentanil (μg)	126 (96,168)	105 (70,163)	0.043
number of times propofol was added	1 (1,2)	0 (0,1)	0.001

Data are displayed as the mean ± SD, or median (interquartile range) and 25th and 75th percentile (p25, p75). One-way analysis of variance or Wilcoxon rank sum test was used to analyze differences between groups. A *p*-values or corrected *p*-values of 0.05 were defined as statistically significant.

Abbreviations: Group S, esketamine-propofol/remifentanil group; Group C, propofol/remifentanil group.

### Comparison of child recovery between groups

The evaluation of recovery quality was based on PAED scores and cough scores in both groups in Tables 4, 5. The mean PAED scores at the time of emergence (T = 0 min), 5, 10, 15, and 20 min were significantly lower in Group S compared to those of Group C. At 25, and 30 min, the mean PAED scores were not significantly different between the groups. There were 6 cases of PAED score in Group S, while 13 cases of the PAED score in Group C were > 10 at the time of emergence (T = 0 min). Among them, the 7 children in Group C and the 2 children in Group S received midazolam intravenously with 0.07 mg/kg, and the other children were calmed due to the company of their parents. No children were observed with acute exacerbations of cough before the FFB procedure and at the time of enrolment in the study. The cough score was significantly lower in groups S compared to that of group C during recovery at emergence (T = 0 min), 5, 10, 15, and 20 min. The cough score of the children at other times (25 and 30 min) was not significantly different in two groups. Children with severe cough were observed in the PACU and received aerosol inhalation therapy with a face mask after examination, medications including budesonide, ipratropium bromide, salbutamol were included until the cough score was < 2.

**TABLE 4:** The PAED scores of two groups.

Parameter	T_0_ min	T_5_ min	T_10_ min	T_15_ min	T_20_ min	T_25_ min	T_30_ min
Group C	9 (8,14.5)	8 (6,10)	6 (5.25,8)	5 (4,6)	4 (2.25,6)	2 (0,4)	2 (0,4)
Group S	8 (6,10)	6 (6,8)	5 (4,6)	4 (4,5)	3.5 (0,4)	0 (0,3.75)	0 (0,3)
*p*-value	0.008	0.020	0.004	0.008	0.033	0.140	0.184

Data are displayed as 25th and 75th percentile (P25, p75). Wilcoxon rank sum test was used to analyze differences between groups. A *p*-values or corrected *p*-values of 0.05 were defined as statistically significant.

Abbreviations: Group S, esketamine-propofol/remifentanil group; Group C, propofol/remifentanil group.

**TABLE 5:** The cough scores of groups.

Parameter	T_0_ min	T_5_ min	T_10_ min	T_15_ min	T_20_ min	T_25_ min	T_30_ min
Group C	3 (2,3)	2 (1,3)	1 (0.25,2)	1 (0,1)	1 (0,1)	0 (0,0)	0 (0,0)
Group S	2 (1,2)	1 (1,1)	1 (1,1)	1 (1,1)	0 (0,0)	0 (0,0)	0 (0,0)
*p*-value	0.001	0.001	0.037	0.044	0.031	0.127	0.370

Data are displayed as 25th and 75th percentile (p25, p75). Wilcoxon rank sum test was used to analyze differences between groups. A *p*-values or corrected *p*-values of 0.05 were defined as statistically significant.

Abbreviations: Group S, esketamine-propofol/remifentanil group; Group C, propofol/remifentanil group.

### Incidence of adverse events

Adverse events are listed in Table 6. The number of injection pains varied greatly between the two groups. The incidence of injection pain was higher in Group C (69.4%, *p* = 0.000) than in Group S (13.9%). No significant differences were found in the incidence of mild injection pain between the two groups (*p* = 0.710). The percentage of patients with moderate injection pain was lower in Group S (2.8%) compared to Group C (25%, *p* = 0.006). The percentage of patients with severe injection pain was lower in Group S (2.8%) compared to Group C (30.6%, *p* = 0.002). There were no significant differences between the two groups in terms of the incidence of laryngospasm and bronchospasm. There are no paradoxical agitation in both groups following midazolam administration. None of the children experienced PONV, vertigo, or hallucinations in either of the two groups.

**TABLE 6:** Comparison of incidence of adverse events between the two groups.

Parameter	Group C	Group S	*p*-value
Patients with pain (*n%*)	25 (69.4%)	5 (13.9%)	0.000
The severity of pain (*n%*)			
No injectin pain	11 (30.6%)	31 (86.1%)	0.000
Mild injection pain	5 (13.9%)	3 (8.3%)	0.710
Moderate injection pain	9 (25%)	1 (2.8%)	0.006
Severe injection pain	11 (30.6%)	1 (2.8%)	0.002
Laryngospasm (*n%*)	6 (16.7%)	1 (2.8%)	0.107
Bronchospasm (*n%*)	1 (2.8%)	0	1.000
paradoxical agitation following midazolam administration	0	0	-
PONV **(** *n* ***%*)**	0	0	-
Vertigo	0	0	-
Hallucination	0	0	-

Data are presented as the number of cases. Chi-square tests or Fisher’s exact test was used to analyze the incidence of adverse events between the two groups

Abbreviations: Group S, esketamine-propofol/remifentanil group; Group C, propofol/remifentanil group.

## Discussion

In this study, we found that the use of esketamine in subanesthetic dose (0.3 mg/kg) combined with propofol/remifentanil would produce a lower incidence of hypoxemia and more stable hemodynamics, lower propofol and remifentanil doses, better recovery from anesthesia, reduced PAED scores, cough scores, and injection pain than standard propofol/remifentanil methods, suggesting that this new sedation regimen may have better prospects for children undergoing FFB.

Combinations of sedatives and opioids are commonly used. However, interactions and synergism between sedatives and opioids can result in oxygen desaturation and fluctuation in circulation, which is a main concern regarding sedation during FFB (Du Rand et al., 2013). Children have a different respiratory physiology than adults; their functional residual capacity of the lungs is slightly lower, while their alveolar ventilation is significantly higher; additionally, oxygen consumption in children is higher. These physiological peculiarities result in a higher risk of desaturation. In recent years, evidence has shown that a subanesthetic dose of esketamine combined with propofol and opioids, reduces the adverse effects of respiratory circulation (Trimmel et al., 2018). It can be seen from this study that the incidence of oxygen desaturation in our Group S was lower than in Group C. The respiratory rate increased at T_2_-T_4_ in Group S than in Group C, while PetCO_2_ in Group S was lower than that in Group C immediately after the end of bronchoscopy, showing that a proportion of children in Group C experienced significant alveolar hypoventilation as indicated by CO_2_ retention. This is attributed to the fact that subanesthetic doses of esketamine reduced the consumption of propofol and remifentanil in Group S, thus reducing the incidence of respiratory depression. Jonkman et al. found that esketamine was effective against remifentanil-induced respiratory depression, an effect due to increased ventilatory CO_2_ chemosensitivity reduced by remifentanil (Jonkman et al., 2018). Our present study further supported earlier preliminary findings. In the present study, we collected values before sedation (T_0_), after sedation (T_1_), immediately after the end of the bronchoscopy (T_0min_), 5 min after the end of the bronchoscopy (T_5min_), and 10 min after the end of the bronchoscopy (T_10min_); however, the two time points when the fiberoptic bronchoscopy reached the glottis and when alveolar lavage was performed were not collected because sampling could be influenced by when the bronchoscope was inserted through the other nostril, which decreased the free volume of the airways. Measures taken to recover from oxygen desaturation using a stepwise airway assist maneuver (increase in oxygen flow, mandibular support, face mask use, and manual ventilation) were also beneficial in preventing the occurrence of excessive hypoxia to some extent.

In the present study, hemodynamic variables including HR, SBP, and DBP in Group S were more stable compared to Group C during FFB into the glottis and alveolar lavage fluid, which stimulated respiratory smooth muscle activity. In this case esketamine, as an antagonist of the NMDA receptor, has sedative, analgesic, and anesthetic effects (Yang et al., 2022). Meanwhile, the analgesic effect of esketamine is promoted by the release of endogenous opioid peptides, which activate opioid μ, δ, and κ receptors, and block sodium and potassium plasma channels (McMillan and Muthukumaraswamy, 2020). On the other hand, esketamine also exerts a sympathetic excitatory effect that counteracted the cardiovascular inhibitory effect of relatively high doses of propofol (Zhan et al., 2022).

The BIS is defined by digital processing and quantification of processed signals obtained from multiple bispectral analysis of the EEG, which is widely used to monitor the depth of sedation during anesthesia (Oliveira et al., 2017). It can prevent intraoperative awareness caused by excessive anesthesia or insufficient medication. At present, data on BIS values in the clinic are mainly derived from propofol, benzodiazepines and inhaled anesthetics, while the calibration of esketamine is not clear. This is mainly related to the effects of drugs on different brain waves. Propofol, benzodiazepines and inhaled anesthetics act primarily on the γ-aminobutyric acid (GABA) receptor, which inhibits excitatory neurons and acts on α and γ waves of the EEG, and has a good correlation between their sedative effect and BIS value. While esketamine acts as an antagonist of the NMDA receptor and mainly activates the θ, δ, and γ waves of the cerebral cortex (Vlisides et al., 2017). The power spectral density of *α* wave decreased and the γ wave increased after the subanesthetic dose of esketamine was administered, which may be related to the acquisition of excited EEG (Nunes et al., 2011). In adults who undergo gastroscopy, the BIS value has also been shown to be significantly higher in those who received propofol combined with ketamine than in patients who received propofol combined with dexmedetomidine (Tekeli et al., 2020). Rogean et al. found that the levels of sedation after the administration of low doses of esketamine correlate with variations in potency of electromyographic activity (elevations), therefore elevating the BIS values (Nunes et al., 2011). The same results were also obtained in this study. Overall, the results of this study showed that the BIS value of Group S at T_1_–T_4_ was significantly higher than that of Group C. This result indicated the ability of BIS to predict sedation/anesthesia levels with esketamine was limited. Patient reactions, such as fluctuations in hemodynamic variables fluctuations (HR, SBP, and DBP) and physical movement, should also be monitored.

In terms of the quality of recovery was defined by the Pediatric Anesthesia Emergence Delirium (PAED) scores and cough scores. Stimulation within the lung by FFB can cause sensations of pain, irritation, and the urge to cough (Irwin, 2006). Severe cough during emergence from anesthesia causes discomfort and it is not conducive to rapid recovery in children (Ozturk et al., 2016). PAED scores has been proved to be the most reliable and authentic in evaluating the appearance of agitation and the degree of pain during recovery because it is difficult for young children to express their experience (Sikich and Lerman, 2004). In the present study, we found differences between the incidence of PAED and cough scores between the two groups. PAED scores were lower at T_0 min_-T_20 min_ in Group S than in Group C. There are many reasons for the agitation during the recovery period, postoperative pain or discomfort is the main factor in emergency irritation. Esketamine can help alleviate the degree of agitation and pain after the children have awakened from anesthesia. The probable underlying mechanisms of the good analgesic effects of esketamine derived from its conversion to norethindrone, which is pharmacologically active and has an anesthetic potency equivalent to 1/5 to 1/3 that of esketamine, but with a longer elimination half-life *in vivo* and mainly by metabolism by liver microsomal enzymes (Zhu et al., 2022). Therefore, esketamine could provide long-term sedation and analgesia, and good sedation and analgesic effects (Zhong et al., 2018). In our study, the time of bronchoscopy was relatively short; thus, the effects of esketamine may have been maintained up to the end of procedure. Furthermore, it can also antagonize the NMDA receptor, relax bronchiolar muscle activity, suppress bronchial constriction due to histamines, and reduce tracheal and bronchial muscle spasms, which all contribute to effectively reduce the incidence of postoperative cough in children and are conducive to recovery (Lu et al., 2021). Qi et al. also found that esketamine decreased the incidence of emergency agitation in children undergoing tonsillectomy without increasing postoperative side effects (Li et al., 2022a).

Regarding recovery time, in our study, we found that the recovery time of the children in Group S was slightly prolonged compared to Group C, although the difference was statistically significant, the average awakening time was only 2 min longer than in Group C, and the longest recovery time of the children in the Group S was 19 min. Furthermore, In the present study there was no postoperative delayed recovery in either group (the recovery time after anesthesia was more than 2 h), the time to the ward from the recovery room were similar between the two groups. Therefore, esketamine did not result in prolongation of the resuscitation.

Qi et al. showed that subanaesthetic dose of esketamine used as an adjuvant in general anaesthesia delays time to eye opening, but it did not result in extension of the time to discharge from the PACU. (Li et al., 2022a). Their findings agreed with those of our present study. However, some other studies have reported different results. Eberl et al. (2020) show that 0.15 mg/kg esketamine may not affect the recovery time of propofol sedated patients during ERCP in patients with ASA scores of I and II, compared with 2 μg/kg alfentanil. One reason for this difference was related to different doses of esketamine used in different studies. On the other hand, which was associated with the sedative effect of esketamine. As with previous studies, PAED scores associated negatively with the child’s time to awakening (Ozturk et al., 2016). Esketamine’s effect on delirium during emergence to 20 min of recovery benefits to our management of these children.

Injection pain is one of the most common adverse reactions of propofol. The overall incidence of injection pain in adults is approximately 50%–80% and can be as high as 90% in children (Bakhtiari et al., 2021). Studies have shown that injection pain caused by propofol infusion will bring an unpleasant anesthesia experience to patients, especially for children, which will further aggravate tension and fear. It is mainly due to the direct stimulation of propofol on afferent peripheral nerve fibers in the inner wall of venous blood vessels. NMDA receptors are expressed in the primary afferent unmyelinated terminals that innervate the peripheral skin. Esketamine has been shown to have a local anesthetic action and additive hypnotic effect by acting on NDMA receptors in the vascular endothelium or central nervous system (Xu et al., 2022). Li et al. (2022b) reported that pre-administration of a subclinical dose of esketamine could effectively alleviate injection pain caused by propofol infusion in adult. Consistent with the results of previous research, Group S was administered an intravenous injection of 0.3 mg/kg of esketamine for the induction of anesthesia, which reduced the occurrence of pain from the injection of propofol in our study.

Furthermore, we evaluated the safety of esketamine in pediatric anesthesia after FFB. No difference was found in the incidence of Laryngospasm, bronchospasm and PONV. Psychotropic effects such as vertigo and hallucination which we found were not present in any of the study participants. Yin et al. (2019) reported that the combined use of ketamine and propofol was beneficial in reducing the incidence of psychotropic symptoms. Yang et al. (2022) showed that the combination of 0.5 mg/kg esketamine and propofol in gastrointestinal endoscopy did not exert any psychogenic effects. Our results provide further validation of these findings.

### Study limitations

There were some limitations to our study that should be considered. First, we did not establish different doses of esketamine in Group S. This study only evaluated 0.3 mg/kg of esketamine combined with propofol and remifentanil in FFB procedures. This is an effective dose but does not fully represent the optimal dose. Consequently, we could not evaluate the dose-response relationship of esketamine combined with propofol and remifentanil as a preoperative period drug. Second, esketamine exerts cognitive side effects. This study only observed postoperative PAED of children and psychotropic effects in the recovery room but did not follow the influence of esketamine on children’s postoperative long-term cognitive functioning. The next step will be to observe the influence of esketamine on postoperative cognitive behavior in children. Third, in this study, the influence of age and gender differences on the results was not considered. Four, this study was a single-centre study with a small cohort. Therefore, the size of the study group would be difficult to increase or homogenize in a single centre trial setting. A larger patient sample and multicenter study are needed to investigate the effects of esketamine on analgosedation.

## Conclusion

We recommend a combination of intravenous 0.3 mg/kg esketamine as an adjuvant to propofol/remifentanil analgosedation and spontaneous respiration for children undergoing FFB. Our study showed that this regimen resulted in a lower incidence of hypoxemia and more stable hemodynamics, less propofol and remifentanil dose requirement, better recovery from anesthesia, as well as reduced PAED scores, cough scores, and injection pain.

## Data Availability

The original contributions presented in the study are included in the article/supplementary material, further inquiries can be directed to the corresponding authors.
